# Stereotactic Body Radiotherapy (SBRT) for an Extracranial Arteriovenous Malformation of the Pelvis

**DOI:** 10.7759/cureus.18750

**Published:** 2021-10-13

**Authors:** Alborz Jooya, Martin E Simons, Derek S Tsang

**Affiliations:** 1 Radiation Oncology, The Ottawa Hospital, Ottawa, CAN; 2 Radiology, Toronto Western Hospital, Toronto, CAN; 3 Radiation Oncology, Princess Margaret Cancer Center, Toronto, CAN

**Keywords:** extracranial, avm, arteriovenous malformation, sabr, sbrt, stereotactic body radiotherapy

## Abstract

Extracranial arteriovenous malformations (AVMs) are rare pathological, benign conditions that are characterized by aberrantly connected arteries and veins without normal intervening capillary vasculature. Although stereotactic radiosurgery is an established, efficacious, safe treatment for intracranial AVMs, there is no known published data on the use of stereotactic body radiotherapy (SBRT) for the treatment of abdominopelvic AVMs.

One patient with an extracranial AVM in the pelvis that was only partially responsive to embolization was treated with SBRT to a dose of 21 Gy, delivered in three fractions over six calendar days.

At presentation, the patient was non-ambulatory due to neuropathic pain from a sciatic impingement of the AVM. The patient underwent two prior catheter-based embolization procedures that had achieved partial obliteration, but with the persistence of neuropathic pain and symptoms. After SBRT to the pelvic AVM, the patient had marked improvement in pain over 10 months and was able to ambulate again. Follow-up angiography and CT demonstrated the obliteration of previously visualized AVM.

We describe the first known report of pelvic AVM successfully treated with a combination of embolization and SBRT. Three-fraction SBRT to a total dose of 21 Gy appears to be safe and effective for extracranial AVMs arising in the pelvis. This strategy may be considered for patients with pelvic AVMs that are refractory to standard interventional therapies. However, these findings should be validated in larger cohorts.

## Introduction

Arteriovenous malformations (AVMs) are rare vascular, benign lesions that are characterized by structural abnormalities of the vascular system with aberrantly connected arteries and veins without normal intervening capillary vasculature [1]. These vascular lesions can arise anywhere in the body and can involve one or more anatomical regions and grow via vascular recruitment and collateralization [1-3]. Population-based studies have estimated the prevalence of intracranial AVMs to be approximately 15-18 per 100,000 persons. However, the prevalence and incidence of extracranial AVMs are less clear, but they are estimated to be 20 times less common [4,5]. Extracranial AVMs are often present at birth and may grow gradually, eventually leading to the development of symptoms and complications with age [2,3,6]. Since veins are incapable of maintaining high blood flow, structural remodeling in response to altered hemodynamics of AVMs leads to their anatomic expansion. The resultant pathological changes of AVMs can lead to serious complications including ischemia, pain, tissue degradation, and structural deformation [3,5-7].

Management of extracranial AVMs remains challenging, and treatment options include endovascular embolization, surgical excision, or a combination of both, and lack of response to initial traditional therapies is associated with poor prognosis [7-9]. Stereotactic radiosurgery, an effective treatment option for intracranial AVMs, is not available for extracranial AVMs because the traditional technique of using a gamma knife device is not possible for targets outside the head. However, hypofractionated stereotactic body radiotherapy (SBRT) has been successfully used in a small number of patients with extracranial AVMs, most with lesions in the head-and-neck region [9-12].

In this case report, we describe a patient with a pelvic AVM successfully treated with SBRT.

## Case presentation

Methods

A retrospective chart review was performed to collect clinical history, imaging, radiotherapy plan, and follow-up details for one patient treated with embolization and SBRT for a pelvic AVM. Because this was a case report describing the experience of a single patient, it was not considered research as per the Tri-Council Policy Statement: Ethical Conduct for Research Involving Humans (TCPS 2) and was exempt from research ethics board review [13]. The patient provided express, written consent for the publication of this case report.

Case description

A 60-year-old woman initially presented with a four-month history of progressively worsening severe radicular left leg pain, most notably in the L5 distribution, weakness, and paresthesia. The pain was described as shooting and sharp in nature, and aggravated by walking, sitting, and bending forward. She was non-ambulatory, largely confined to a wheelchair due to the pain, and a one-person assist with toileting and transfers. The patient manifested no other symptoms at that time, and the remainder of her physical examination was unremarkable. Her past medical history was significant for asthma, bronchiectasis, and chronic obstructive pulmonary disease (COPD).

**Figure 1:**
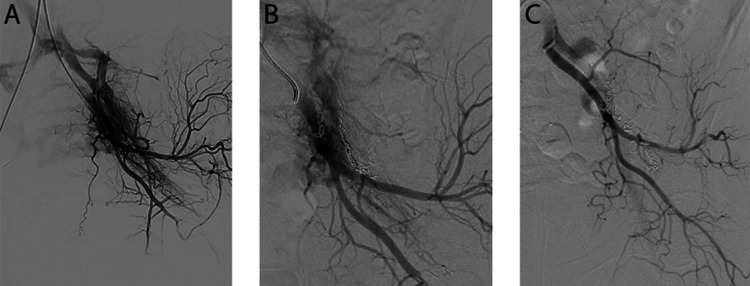
Spinal arterial angiography of the left internal iliac artery. (A) Pre-embolization showing diffuse arteriovenous malformation. (B) After two embolization procedures one month apart. (C) Post radiotherapy (10 months after SBRT). SBRT: Stereotactic Body Radiotherapy

She did not find any pain relief from exercises recommended by physiotherapists and a chiropractor under the presumptive diagnosis of sciatica. As part of further workup, she underwent MRI of the spine, which was suspicious for an arteriovenous fistula in the lower lumbar and sacral region, specifically between the left gluteus maximus and gemellus muscles with supply from a branch of the left internal artery and venous drainage to the left internal iliac vein. Sacral and pelvic angiography demonstrated an AVM arising from the left internal iliac artery with significant shunting into dilated presacral veins (Figure 1A).

Moreover, the malformation completely surrounded the sciatic nerve, with associated enlargement of its vasa nervorum.

**Figure 2:**
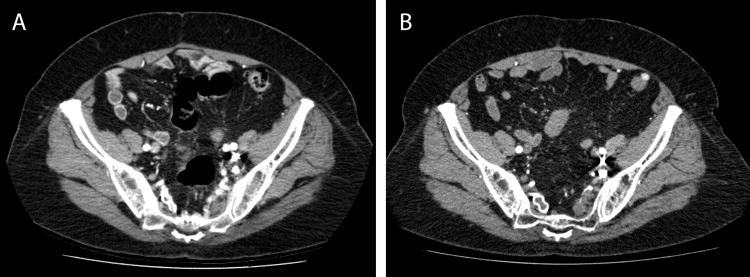
Contrast-enhanced CT of the pelvis, before (A) and 10 months after SBRT (B). On the post-radiotherapy study (B), note the persistence of bright embolization material along the left pelvic sidewall anterior to the left sacrum, but the resolution of enhancing AVM nidus and surrounding vessels.

The patient was then referred to interventional radiology service, and she underwent two embolization procedures with NBCA glue (Glubran, GEM Srl, Viareggio, Italy), targeting branches of the internal iliac artery and vein; the embolizations were set one month apart (Figure 1B). The patient noticed a partial improvement in her neuropathic pain after each subsequent embolization; however, given persistent symptoms, she was referred to radiation oncology service for further management. Diagnostic contrast-enhanced CT post-embolization confirmed residual AVM (Figure 2A).

Management with radiotherapy

**Figure 3:**
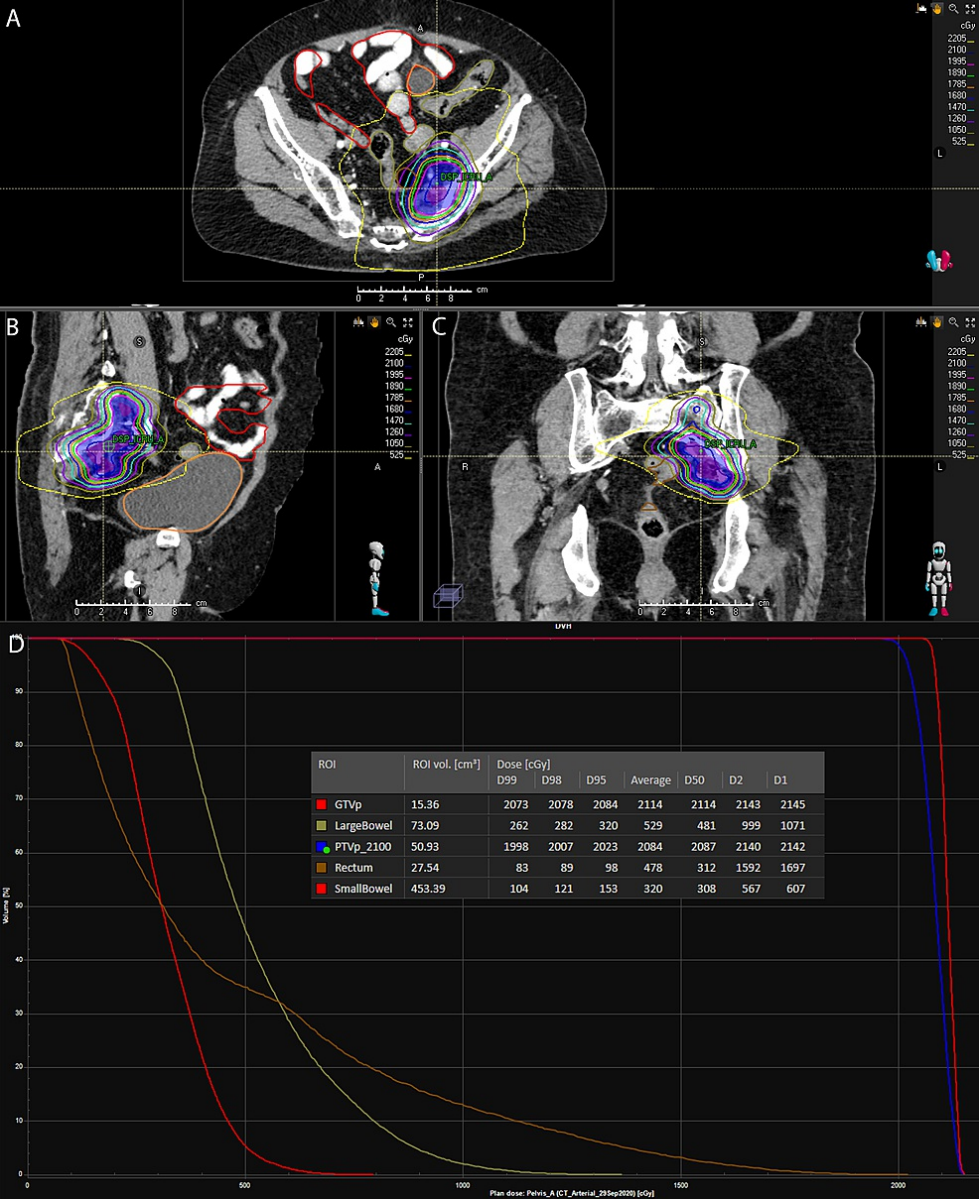
(A) Axial. (B) Sagittal. (C) Coronal sections of the delivered conformal stereotactic body radiotherapy (SBRT) plan using 6 MV photons. (D) Dose-volume histogram (DVH) and dose statistics for the target and OARs. Dx represents the x% of the specified volume receiving at least the displayed dose.

After a thorough informed-consent discussion, emphasizing the rarity of pelvic AVMs, and limited data surrounding the use of fractionated radiotherapy for its management, the patient voiced understanding and agreed to receive SBRT. Instructions were provided regarding daily bowel preparation to minimize rectal volume. She underwent computed tomography (CT) simulation in the supine position with a soft mattress. To assist in target volume delineation, intravenous iodinated contrast material was administered and arterial (30-second delay) and venous (90-second delay) phases were obtained. The target volume was 15.4 cm^3^. A single-arc conformal SBRT plan was generated using 6 MV photons, to be delivered via volumetric modulated arc therapy (VMAT) (Figures 3A-3C and Video 1). A total dose of 21 Gy in three fractions (7 Gy per fraction, every other day, over six calendar days) was prescribed. An expansion of 0.5 cm on the treatment volume was used to create the planning target volume (PTV, 50.9 cm^3^). This dose was chosen to minimize the risk of sacral plexopathy, based on a clinical trial guideline to keep the sacral plexus D5cc <21.9 Gy with a three-fraction regimen (ClinicalTrials.gov identifier, NCT03721341). With respect to coverage, 95% of PTV received 20.23 Gy, while dose to the rectum was minimized (rectum Dmax = 20.22 Gy, D0.1cm^3^ = 18.04 Gy, D1cm^3^ = 14.6 Gy (Figure 3D). Image guidance with cone beam CT scan on each treatment day was used to confirm proper set up prior to each treatment, with a match to bony anatomy and radio-opaque embolization coils. The patient completed treatment without any observed toxicity.

**Video 1:** Axial sections of the delivered SBRT plan, with isodose distributions from the treatment planning software (RayStation).

Follow-up five months after SBRT, the patient reported a slow, gradual decrease in her neuropathic pain, without evidence of sciatic nerve injury after radiotherapy. Contrast-enhanced CT and catheter angiography 10 months after SBRT demonstrated complete resolution of AVM following radiotherapy (Figures 1C, 2B). Upon clinical assessment approximately 11 months post radiation treatment, the patient reported marked improvement in her left leg pain, paresthesia, and weakness; she was now ambulating independently without gastrointestinal or genitourinary toxicities.

## Discussion

AVMs are rare vascular lesions with aberrantly connected arteries and veins that can arise in the central nervous system, and less commonly extracranially. The continual growth of AVMs can lead to focal destruction of underlying tissue, which could, in turn, lead to various symptoms, including pain [3]. The management of intracranial AVMs is well-established, and it often includes surgery or stereotactic radiosurgery (SRS) [1]. In contrast, extracranial AVMs are rare, and their treatment is less standardized. Management options include endovascular embolization with or without subsequent surgical resection [5,8,14]. Extracranial AVMs are frequently treated with multiple embolizations; however, given high rates of recurrence, radiation treatment may be considered [5]. Successful management of pelvic AVMs with embolizations has been reported in a number of series [15-23]. Furthermore, a recent systematic review has highlighted the evidence behind the use of stereotactic radiosurgery and fractionated radiotherapy for the management of spinal AVMs, including intramedullary lesions of thoracolumbar junction and conus medullaris [24]. However, to the best of our knowledge, there have been no studies to date describing the use of radiotherapy in AVMs of the abdomen or pelvis, and this report is the first case of a pelvic AVM treated with fractionated SBRT.

Radiation targets endothelial cells, and also induces progressive thrombosis and eventually obliteration of vessels [25]. The likelihood of success of radiotherapy is dependent on the dose delivered to the nidus, with a 60% to 70% obliteration rate for single-fraction margin doses of 15-16 Gy, and more than 90% for margin doses of 20-25 Gy [26,27]. Cerebral AVMs are typically managed with high-dose single-fraction SRS, which has been shown to be safe and effective; however, this approach is not possible in the pelvis, as the risk of gastrointestinal or neurologic toxicity would be too high with single-fraction radiation treatment. Assuming a simple linear-quadratic model [28] and a/b = 2, a course of 21 Gy in three fractions is approximately equivalent to a single-fraction dose of approximately 13.1 Gy, which is less than the minimum standard single-fraction SRS dose for intracranial AVMs (15 Gy). However, there is some controversy surrounding the radiobiological therapeutic gain of hypofractionated regimens for large AVMs, as a majority of studies that have investigated the vascular radiobiological effects of high-dose per fraction focused on cancerous tumors that may have different pathogenetic and radiobiologic characteristics from AVMs [29,30]. Studies of stereotactic radiotherapy for the management of spinal AVMs have considered SBRT doses of 20 to 21 Gy over two to four fractions to be acceptable for the spinal cord [24,31]. Our SBRT dose was limited by a clinical desire to minimize the risk of sacral plexopathy and injury to the sciatic nerve, and previously outlined guidelines were used (ClinicalTrials.gov identifier, NCT03721341). There is a small volume of published data on the use of fractionated stereotactic radiotherapy to achieve successful treatment of extracranial AVMs of head and neck and extremities [9,11,12], summarized in Table 1.

**Table 1:** Selected summary of case reports describing hypofractionated radiotherapy for AVMs outside the central nervous system.

Study	Location	Target volume (cm^3^)	Total dose	Fractions	Technology	Outcome	Time to best response
Jooya et al. (this study)	Pelvis, sidewall	15.4	21 Gy	3	Linear accelerator (SBRT)	Obliteration	10 months
Koyfman et al. [12]	Infratemporal fossa; tongue	177	24 Gy	3	Linear accelerator (SBRT)	“Dramatic reduction”	54 months
Trombetta et al. [9]	Thigh, lateral	15 cm region	30 Gy	5	Linear accelerator (3DCRT)	Obliteration	12 months
Saito et al. [13]	Tongue	130	22 Gy	2	CyberKnife	Obliteration	21 months

## Conclusions

Management of extracranial AVMs remains challenging; SBRT is a safe and effective treatment modality for extracranial vascular malformations of the pelvis and can yield excellent results in selected cases. We propose that radiotherapy using high doses per fraction is effective for the obliteration of the nidus, but it needs to be balanced with minimizing the risk of toxicity to the surrounding tissues, specifically sacral nerves, and bowel. We report a single patient successfully treated with SBRT without any reported toxicity; however, to establish efficacy, larger studies may be needed. This strategy should be considered as a safe and effective treatment modality for patients with challenging extracranial AVMs that are refractory to standard interventional therapies and holds promise for long-term symptom control with minimal associated morbidity.
